# Lessons Learned on the Use of Wearables in Tracking Health Indicators Among Farmworkers in North Carolina Exposed to Extreme Heat Conditions

**DOI:** 10.1002/puh2.70294

**Published:** 2026-06-09

**Authors:** Deshira D. Wallace, Vidal Lordmendez, Sarah A. Perry, Laura Villa Torres, Lindsay Savelli, Melissa Castillo, Yesenia Cuello

**Affiliations:** ^1^ Gillings School of Global Public Health University of North Carolina at Chapel Hill Chapel Hill North Carolina USA; ^2^ NC FIELD Goldsboro North Carolina USA

**Keywords:** climate change, farmworkers, heat, Latino, public health, wearable devices

## Abstract

**Introduction:**

Farmwork is a hazardous occupation that is particularly vulnerable to the impacts of climate change. Data collection methods are needed to understand the hazards affecting farmworkers. The objective of this study is to share lessons learned regarding the feasibility and acceptability of using wearable technology to assess the role of heat exposure on health indicators among farmworkers in Eastern North Carolina.

**Methods:**

All participants were Latino men farmworker adults (18+ years) working in Eastern North Carolina who wore a wearable health tracker for 90 days. The participants were asked to wear the device day and night to collect information on heart rate, blood oxygenation, skin temperature variation, breathing rate, sleep, and stress. Participants were recruited with a community partner across six counties in the region.

**Results:**

Existing community relationships with farmworkers facilitated adoption of new technologies. The participants found the wearables acceptable and easy to use. Wearable feasibility was dependent on consistent broadband internet for data uploads; however, broadband availability was low (35.7%–71.1%) across counties. The vendor's infrastructure (i.e., device and app) limited who could be recruited for the study. Climate impacted the expected length of study participation as droughts and floods cut the growing and harvesting season short, which impacted data collection.

**Conclusions:**

Testing and consideration of the structural strengths and constraints are needed for selection of data collection instruments. Research protocols need to be nimble to guarantee successful data collection for climate‐related research with minoritized workers such as farmworkers in the United States.

## Graphical abstract

We assessed how farmworkers fared wearing a wearable device over the summer months. The smartwatch adoption was high but not consistent over 90 days. The use of the related app was low. Challenges for use were inconsistent internet and the intersection of immigration policies and climate change.

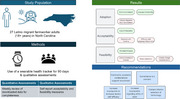

## 1 Introduction

Agriculture, one form of outdoor work in the United States (US), is a multibillion‐dollar industry in North Carolina and is the top industry in the state [1]. The industry is heavily dependent on a workforce from Latin America. Of the almost 75,000 farmworkers who responded to a North Carolina Department of Commerce survey in 2021, 74% are undocumented or legal permanent residents from Latin America (i.e., seasonal farmworkers), and among all farmworkers, 32% arrive in North Carolina under the H‐2A temporary agricultural workers visa program (i.e., migrant farmworkers) that allows US employers to fill temporary agricultural jobs with workers from abroad [2]. Although nationally, 75% of hired farmworkers report preferring communicating in Spanish, in North Carolina, it is 94%, indicating further needs to ensure responsive and language‐accessible research [3].

Notably, agriculture is one of the most hazardous industries in the labor market. Farmworkers are overexposed to a range of chemical and climate‐related hazards [4]. For example, the rate of occupational heat‐related deaths among Latin American and Latino (henceforth referred to as Latino) workers (2.21 per 1,000,000 person‐years) is over 11 times that of non‐Latino workers in North Carolina [5]. The rurality of farmworker communities further complicates access to cellular services, broadband internet, healthcare, and emergency management services to address climate‐related exposures [6].

Most agricultural activity in the state occurs in Eastern North Carolina, making it an ideal location to explore farmworker experiences. In Eastern North Carolina, workplace conditions in the event of extreme heat exposure have led to cases of heat‐related illness [7, 8]. However, there is little documented evidence of the level of exposure and related outcomes for this population [9]. Much of the difficulty in understanding the climate‐change‐related exposures and related outcomes among farmworkers is tied to data collection methods [10, 11]. Qualitative studies provide depth on experiences; however, participants may not always attach climate exposures, such as heat, to physiological and psychological changes they experience, making it difficult to examine causality. Alternatively, quantitative studies among outdoor workers, including farmworkers, use a variety of measures to assess heat exposure, including documenting daily temperatures and wet‐bulk global temperature [11]. Physiological measures include body temperature, heart rate, metabolic work rate, body mass, blood pressure, and kidney function using urine and blood tests during the working season [11]. Data collection strategies often involve self‐report, which provides essential data; however, self‐report is prone to recall bias [8, 12, 13].

To address concerns about exposure and subsequent physiopsychological responses and take advantage of new technologies, there has been a rise of the use of biosensor technologies, including wearable health trackers for research. The use of commercial trackers (e.g., Fitbit and Garmin) for real‐work data collection has been more extensive since 2011 [14]. Although there are studies that used technologies to assess physiological changes among Latinos in the US [15], data are still lacking for outdoor workers in rural settings. On the basis of published articles, these technologies have focused on urban settings with access to broadband internet [14]. Few published papers have demonstrated the feasibility and acceptability of using wearable health trackers among farmworkers or similar rural, migrant populations who engage in outdoor work. A recent scoping review of 19 global studies on the use of wearable devices among heat‐exposed outdoor workers noted only five studies focused on farmworkers. Therefore, a gap remains on the effective use of wearable technologies for rural farmworkers [16], and if farmworkers, who are under‐engaged in research and not English speakers, appreciate this data collection method.

Therefore, our community‐engaged study utilized the Framework for Developing and Evaluation Complex Interventions (hereafter “Framework”) [17] to assess the feasibility and acceptability of a pilot intervention using wearable health trackers among farmworkers engaged in agricultural labor in Eastern North Carolina, a rural region of the state with limited broadband internet access.

## 2 Methods

### 2.1 Community Engagement

Aligned with community‐based participatory research principles, our team comprised an existing academic‐community partnership who co‐designed a 90‐day observational study to examine the effects of excess heat on cardiovascular and psychosocial risk factors among farmworkers [18]. The initial study idea came from NC FIELD, the community partner, who has worked with rural farmworker communities for over a decade and comprises staff who have lived experiences as migrant and seasonal farmworkers. The study design was adjusted over time by both NC FIELD and the University of North Carolina at Chapel Hill (UNC) faculty, who have Latin American and immigrant backgrounds.

To ensure balance between research and action across organizations, the study team comprised members of both NC FIELD and UNC, who co‐developed the study protocol and trained together on human subjects research ethics through a series of trainings, including Collaborative Institutional Training Initiative (CITI) or CIRTification (for community partners) training prior to research initiation. Community partners appreciated the CIRTification training as it provided the team with foundational language and understanding of how to engage in research while accounting for community needs. We disseminated finding in Spanish so the broader community could respond to results and provide feedback.

### 2.2 Study Design

Recruited and verbally consented participants were asked to wear a Fitbit for 90 days all day and night and upload their data at least every 7 days. The data presented for this study are focused on the wearable utilization data and reflections of participants on their acceptability of the wearables as a means of data collection. Participants received $40 and kept the Fitbit Inspire 3 upon completion of the study.

### 2.3 Participants

Inclusion criteria were (1) being an adult (18 years and older); (2) being a migrant (H‐2A) or seasonal farmworker; (3) working in Eastern North Carolina; (4) self‐reporting a Latin American migrant or US‐born Latino background; and (5) consenting to participate. As over 80% of farmworkers in North Carolina self‐identity as men [19], we focused recruitment on men. All participants were recruited from Eastern North Carolina, a region with high levels of agricultural productivity. Farmworkers in this region harvest crops, such as tobacco, corn, sweet potatoes, berries, melons, and tobacco, which are all harvested by hand. Farmworkers work on average 10–12 h per day, 6 days a week. NC FIELD led recruitment due to established rapport, which facilitated recruiting first‐time research participants.

### 2.4 Data Collection

Data were collected between July and October 2024. We collected individual data from the Fitbit Inspire 3 wearable health trackers worn during the day and night. We collected a range of information, including heart rate, blood oxygenation, steps, sleep tracking, and a composite stress score derived from device measures. Participants were asked to download the Fitbit application (app) on their phone, pair the device, and complete an orientation of the app. Our team supported technical assistance (app download, pairing, and orientation) after in‐person consent and device delivery, thereby ensuring that all participants had a paired device at the start of the study. All participants were primarily Spanish speakers with a variety of literacy levels; thus, all instructions were provided verbally in Spanish.

Monitoring the use of wearables involved weekly review of exported .csv files and in‐person participant responses to open‐ended questions about their ease of using the wearable health trackers and the app. The study team took field notes on the recruitment process for discussion throughout the analysis process.

### 2.5 Data Analysis

We used the Framework to examine the complexity of the pilot, which was adapted to explore the feasibility (i.e., feasibility and acceptability), evaluation (i.e., was intervention the appropriate way to address the research question), and implementation of an intervention (i.e., uptake for health intervention) [17]. The Framework also assesses the bidirectional impacts of these factors accounting for key uncertainties or different settings and contexts [17]. Quantitative outputs included the initial use of the device, evaluation of synced data to assess the number of days the device was used during the 90‐day observation period, and the proportion of participants who completed the 90‐day observation period. Qualitative output data included self‐reported barriers and facilitators for using the wearable health tracker and related app. Additionally, our team debriefed weekly to discuss recruitment and implementation processes to determine strengths and challenges for future scale‐up. We also collected contextual information from the North Carolina government to frame the feasibility of the study in rural settings. Although we collected information‐specific biometric data (i.e., heart rate), the goal of this analysis was to examine the extent to which wearable health trackers can function as a data collection tool for outdoor workers in rural areas.

## 3 Results

The Framework guided organization of results by technology adoption, acceptability, feasibility, and contextual details that impacted study implementation.

### 3.1 Participant Demographics

We recruited 27 farmworkers. All participants were men, between 25 and 50 years old, Spanish speakers, migrant farmworkers, and were all first‐time research subjects. All participants were recruited from farmworker camps (i.e., employer‐provided housing) across six counties in Eastern North Carolina: Bladen, Cumberland, Duplin, Harnett, Hertford, and Wayne.

### 3.2 Adoption

We examined broad adoption, persistence, and utilization. All participants (*n* = 27) used their Fitbit, which indicates high initial adoption. Persistence or the continuous use of the Fitbit for 90 days was not achieved by any participant. On the basis of downloaded data, 30% (*n* = 8) of participants used their Fitbits every day for about 60 days. Regarding utilization, on average participants used their Fitbits for 43 days (range: 4–73 days).

### 3.3 Acceptability

#### 3.3.1 Use and Acceptability of Wearables

As all participants were first‐time research participants, and several described that they were not technologically savvy, we explored how acceptable it would be for farmworkers to use the device. Broadly, participants were amenable to the Fitbit and reported ease of use, as they were familiar with wearing watches, and appreciated how light and discrete the device was, which made it appropriate to wear while engaging in farmwork.

Another component of the acceptability of the Fitbit device revolved around surveillance and privacy concerns. Specifically, there was a concern about tracking and surveillance using the devices’ GPS capabilities. These concerns came about prior to consent in about 41% (*n* = 11) participants; however, during the consent process, we clarified that we were not using GPS capabilities, and location data would not be downloaded. This assuaged any confidentiality and security concerns for all participants. Overall, the Fitbit Inspire 3 was an acceptable wearable health device for farmworkers.

#### 3.3.2 Use and Acceptability of Application

The Fitbit app's English‐language settings made it difficult for participants to navigate. Language changes could only be done on Fitbit.com and not within the app itself, which added difficulties for participants. The study team provided technical support to adjust language settings at participants’ request. Participants mentioned not utilizing the app on a regular basis, unless they were probed to sync their data. Generally, participants did not use the app to explore their health statistics.

### 3.4 Feasibility

#### 3.4.1 Broadband Internet Matters

Internet access was evaluated at two levels, at the county level and at the individual level. Participants were told to sync the device every 7 days. However, almost all farmworkers who reside in employer‐provided housing in Eastern North Carolina do not have access to consistent and reliable broadband internet [20]. In fact, broadband internet adoption ranged from 35.7% to 71.1% for study counties [21].

At the individual level, cellular service is equally inconsistent in these same counties, and employer‐provided housing often did not include internet services. NC FIELD provides hot spots for many farmworker camps to facilitate access to communication streams, such as WhatsApp. As wearable devices require access to the internet to upload data from the device to the app, we selected participants who had internet access. To assess individual‐level internet availability, the study team reviewed weekly data uploads. Weekly uploads versus daily uploads were selected to account for variability in daily internet availability and to assess data storage capabilities of the wearable. Approximately 60% of participants who were actively using their Fitbit devices were able to upload data on a weekly basis.

#### 3.4.2 App Functionality

One form of assessing feasibility of the Fitbit application was to determine what participants needed to create accounts and their comfort in doing so. At the time of the study, Fitbit, now owned by Google, required users to link a personal Gmail account to the Fitbit app for successful data uploads. However, only two of 27 recruited participants had a Gmail account that they could access (i.e., remember their password). Generally, participants had Yahoo accounts or could not recall their email addresses during study recruitment. We asked participants about their comfort with creating and linking new Gmail accounts, and none of the remaining participants (25 of 27) felt comfortable.

To address this concern, we created Gmail accounts and linked each account to the Fitbit app during enrollment to facilitate access, consistency, and confidentiality. The Gmail accounts were specific for study use and served to collect Fitbit data downloads but also served to collect limited identifiable information. However, the bulk creation of study‐specific email addresses by our study team encountered several limitations. First, we could not create Gmail accounts in bulk for research purposes [22, 23], given Google's preference for pairing Fitbit accounts with personal Gmail accounts. Second, creating individual Gmail accounts was also limited by the number of accounts attached to local phone numbers, of which not all participants had given the transnational nature of their lives. Ultimately, logistic barriers requiring Gmail‐specific accounts to support data collection slowed recruitment and caused friction in the acceptability and adoption of wearable technology.

Despite initially activating their Fitbit accounts using the app, difficulties remained for participants to use the app consistently throughout the project period. When we asked why, they reported limited Spanish language in the app made navigation hard, as well as not understanding the utility of logging into the app regularly (i.e., once a week to ensure data uploads) or in reviewing the information stored.

A summary of adoption, acceptability, and feasibility factors is presented in Table 1.

**TABLE 1:** Summary of adoptions, acceptability, and feasibility of Fitbit use.

Domain	Description	Measure	Results
**Adoption**			
(Broad) adoption	Activation and use of Fitbit	Initial activation and use of Fitbit	100% participant adoption
Persistence	Daily use of Fitbit	90 continuous days of use	0% of participants
Utilization	Average number of days of Fitbit use	0–90 days	43 days Range: 4–73 days
**Acceptability**			
Use and acceptance of Fitbit	Use and acceptance of wearable	Self‐reported use and acceptance of wearable	Participants reported Fitbit was easy to use and acceptable for daily use
Use and acceptance of Fitbit (surveillance)	Concerns with GPS tracking of Fitbit device	Self‐reported concerns with surveillance	Pre‐consent: 41% Post‐consent: 0%
Use and acceptance of application	Use and acceptance of app	Self‐reported use and acceptance of app	Low utilization of regular use of app due to limited familiarity and difficulties with shifting app language to Spanish; low understanding of the apps’ utility
**Feasibility**			
Broadband internet (county level)	County‐level broadband internet access for data uploads	North Carolina Digital Equity Division broadband internet county‐level adoption	35.7%–71.1% adoption
Internet availability (individual level)	Participant access to internet (broadband, cellular data, hotspots)	Proportion of participants able to upload data on a weekly basis	60% participant upload
Availability of Gmail accounts	Participants who had a Gmail account and remembered the password for linking to Fitbit app	Self‐reported number of participants who had regular access to a Gmail account	7% (2 of 27) had Gmail access availability
Comfort in creating Gmail account	Participants who felt comfortable creating a new Gmail account and linking to Fitbit app	Self‐reported comfort	0% (0 of 25) felt comfortable creating a Gmail account to link to app
Bulk Gmail account creation	Research‐team created Gmail accounts in bulk	Yes/No	No
Gmail account creation (individual)	Creation of individual email accounts by study team	Yes/No	Yes

### 3.5 Contextual Factors

Adoption, acceptability, and feasibility were impacted by broader contextual factors. One of the primary factors that impacted the execution of the pilot was climate change. Climate change impacted the 2024 agricultural season. Heat waves and drought conditions at the start of the summer season combined with unexpectedly high rainfall during midsummer affected crop yields. Poorer than expected crop yields led some farm owners to reduce farmworker hours or shorten their contracts. Although farmworkers travel within the state, often going from eastern to western North Carolina, Hurricane Helene devastated western North Carolina, thereby reducing work opportunities for farmworkers. H‐2A visa holders were particularly vulnerable to climate change disruptions as shortened contracts meant a return to their home countries in Latin America. We anticipated that a 90‐day observation window would be feasible given past agricultural season trends of May–November. However, climate‐related impacts meant a shorter than average observation window. In fact, farmworker participants, all of whom were H‐2A visa holders, were requested to leave Eastern North Carolina starting early September, some with 1 week's notice, therefore requiring adjustments to the study protocols.

## 4 Discussion

Using the Framework for Developing and Evaluation Complex Interventions, we examined how farmworkers in rural Eastern North Carolina used wearable health trackers. Our participants were Spanish‐speaking, first‐time research participants; thus, partnering with a local, trusted community organization to co‐design a research study was essential for participant engagement. Our results demonstrated that adoption of devices was high with technical support offered by the study team; however, persistence and feasibility varied due to several contextual factors such as internet access and climate change impacts. Additionally, app use was consistently low due to a disconnect of the utility of the app and difficulties with changing the language to Spanish for navigation.

One limitation of our study was the selection of the Fitbit wearable device. Although Fitbits vary in accuracy for different measures, such as underestimating step count and overestimating moderate‐to‐vigorous physical activity, heart rate measures, which were of interest in our work, were deemed to be accurate [14, 24]. Skin tone is a documented concern for accuracy of optical measurements; however, the data are inconclusive on whether darker skin tones over‐ or underestimate measurement [25, 26]. Fitbit, now owned by Google, required users to use a personal Gmail account for the Fitbit account used for data uploading; however, Gmail accounts were not common among our participants, and many did not know how to create one. When we launched the study in 2024, Fitbit was recently transitioning to a Gmail‐only platform. Potential solutions include educating participants to increase their self‐efficacy to use their personal email addresses, seeking alternative devices that allow flexibility on how participants are connected, or using platforms that do not require email addresses for data collection.

As most North Carolina farmworkers are native Spanish speakers [27], the need for language‐appropriate engagement for public health studies is essential. Using a community‐based participatory approach from project design to execution facilitated language accessibility, access to camps, and trust‐building between outside researchers and farmworkers. Although we were able to navigate internet limitations, digital equity is a major concern for engaging with rural farmworker communities. Studies have shown that digital equity, a social determinant, is one of the major hurdles that impact the health of rural populations in the US [28, 29]. In fact, increasing the proportion of adults with broadband internet is a Healthy People 2030 objective [30]. Recent studies on digital equity in rural Eastern North Carolina aligned with our results in that fewer than 25% of households have broadband internet [31]. On the basis of a study of 1034 migrant and seasonal farmworkers in Eastern North Carolina, only 9.8% had internet connection, and 93.9% relied on cellular networks, which can be unreliable [32], highlighting the extent of digital disparities. Encouragingly, the North Carolina state government has a broadband and digital equity division that aims to increase broadband connectivity across the state [21].

When working with rural farmworker communities, research protocols must be nimble to account for research familiarity, limited infrastructure, flexibility of data collection tools, and structural and environmental stressors such as migration‐related polices and climate change. For instance, farmworkers were directly impacted by climate change disruptions, as shown in the results. The climate‐related impacts of 2024 are not unique. North Carolina summers (May 1–September 30), like many areas around the US, are getting warmer, more intense, and longer [33, 34]. Rural areas, especially Eastern North Carolina, are especially vulnerable to climate change impacts due to the high likelihood of engagement in outdoor work [35, 36]. Climate‐change‐related effects, particularly heat, highlight that typical patterns of labor will likely shift, and community groups and researchers need to adjust to those changes as well.

The expansion of wearable devices as data collection tools for rural workers, like farmworkers, remains an important area to explore. With increasing calls to use technologies to support public health research and practice, testing to ensure that technologies function with the population and context of interest is needed. For example, Tan et al. constructed a hyper‐personalized medicine framework (i.e., Healthcare 5.0) featuring the integration of biological, genetic, lifestyle, and environmental data that influence individuals’ health [37]. The goal of Healthcare 5.0 is to provide individualized care that is human‐centric and supports an adaptable healthcare system. One component of Healthcare 5.0 that aligns with the needs of frontline community organizations is having access to data that allow for proactive responses to sudden changes in farmworkers’ health [37], particularly during times with greater vulnerability to heat‐related illness. However, the infrastructure needed for real‐time data collection and monitoring continues to be a concern for rural communities and organizations. Although Tan et al. note privacy and security as a primary ethical concern of Healthcare 5.0 and present data‐related solutions to reduce vulnerabilities (i.e., restricted use data, VPN) [37], they did not address the perception of medical mistrust by certain communities such as Latinos with limited English proficiency [38, 39, 40], which can reduce healthcare engagement.

### 4.1 Recommendations for Future Digital Health Studies

Our results informed recommendations for future digital health studies that are inclusive of communities like farmworkers and other outdoor workers:

1. **Collaborations with trusted partners support recruitment efforts**: Community engagement is essential for public health research, and especially so when recruiting from and working with community members who are underrepresented in research. Fostering co‐design of research with community partners who are trusted in their communities increases the success of project recruitment and implementation.
2. **Surveillance remains a concern**: With the passive data collection, including GPS, a primary concern was surveillance and privacy. Working alongside a community partner allows for anticipation of community‐level concerns and necessary adjustment of protocols. A broader suggestion for working with migrant and seasonal farmworkers, many with vulnerable documentation status, particularly during times of high immigration enforcement, is to consistently re‐evaluate protocols and collect the minimum required information to protect highly vulnerable participants.
3. **Increase touchpoints with participants to ensure understanding of wearable technologies**: Participants may require extended orientation on the use of technologies and the purpose of these technologies in research. The use of wearable devices like smartwatches may have straightforward adoption; however, participants require interactions throughout the study period to improve self‐efficacy of consistent use.
4. **Participants need to understand the utility of apps**: Although wearables, such as smartwatches, are relatively easy to use, navigating related apps is difficult for participants who are unfamiliar navigating similar platforms or if the interface language is not easily changeable. Therefore, early and regular orientations on how to use an app, syncing data (i.e., increase self‐efficacy for data uploading), and the utility of using the app to review health metrics (i.e., self‐efficacy to review health metrics) are needed to improve the engagement with an app.
5. **Context informs the selection of technology**: Although pilot results demonstrated that the use of wearable health trackers can be appropriate for data collection, inequitable access to broadband internet can impact implementation. In rural areas, especially when working with migrant farmworkers who reside in employer‐provided housing and have limited internet access, seek wearable options that have extended data storage (14 days or more) to increase research flexibility.

## 5 Conclusions

Our community‐engaged study evaluated the adoption, acceptability, and feasibility of wearable health trackers and found that although the use of noninvasive and familiar trackers is acceptable for use in Spanish‐speaking and often migrant farmworkers, adoption and feasibility are mixed due to contextual factors, including broadband internet, language‐concordance, and climate‐related effects. Developing programs and research that are nimble and address local contexts in real‐time may be necessary to account for structural changes for research engagement with farmworkers. The development and adaptation of technologies must be tested alongside communities in a variety of environments to improve equitable use.

## Author Contributions

Deshira D. Wallace contributed to the conceptualization of the project and manuscript, lead writing, and editing of the manuscript. Vidal Lordmendez led recruitment and manuscript conceptualization as well as manuscript writing support. Sarah A. Perry supported data collection and writing and editing of the manuscript. Laura Villa Torres drafted the manuscript and provided in‐depth writing editing. Lindsay Savelli provided support with study coordination and reviewed the manuscript. Melissa Castillo and Yesenia Cuello provided manuscript writing support and access to participants.

## Funding

Funding for the University of North Carolina Center for Environmental Health and Susceptibility (CEHS) pilot grants is made possible by support from the National Institute of Environmental Health Sciences (NIEHS): P30ES010126. We also thank the Z. Smith Reynolds Foundation for their support of the UNC Environmental Justice (EJ) Clinic.

## Ethics Statement

The study protocol was approved by the University of North Carolina at Chapel Hill's Institutional Review Board (#24‐0907; Approval date: May 24, 2024).

## Consent

The authors have nothing to report.

## Conflicts of Interest

The authors declare no conflicts of interest.

## Data Availability

The data that support the findings of this study are available from the corresponding author upon reasonable request.
